# Society Meetings

**Published:** 1911-02

**Authors:** 


### SOCIETY MEETINGS

The American Public Health Association will hold its 1911 meeting in Havana, Cuba, from December 4 to 9. The prospect of having the Association again in Havana has aroused the warmest interest among the physicians there, the Secretary of Sanitation, Dr. Varona, being particularly interested. The Academy of Medicine has offered its building for the general section meetings. The Hotel Savilla will be the headquarters of the association.

---

A few years ago a meeting in Havana would probably have discussed yellow fever. The changed situation in Cuba with respect to that disease is shown by the fact that yellow fever has been so completely extinguished on the island that the local physicians desire rather that tuberculosis be given the most prominent place. The question of the milk supply will also be considered. The secretary, William C. Woodward, of Washington, D. C., says it is hoped at this meeting that the recently organized Sociological Section, and the Section on Sanitary Engineering, which was tentatively authorized by the Milwaukee meeting, may be put upon substantial foundations.

---

THE MEDICAL SOCIETY OF THE STATE OF NEW YORK will hold its one hundred and fifth annual meeting Tuesday and Wednesday, April 18 and 19, 1911, under the presidency of Dr. Charles Stover, of Amsterdam. Dr. J. W. Grosvenor, of Buffalo, is first vice-president, and Dr. Wisner R. Townsend, of New York, is secretary. Through lack of proper information the JOURNAL in its issue for January announced this meeting to occur at the usual date—namely, the last Tuesday in January; whereas, at the annual meeting in 1910, the next meeting was appointed for the 3d Tuesday and Wednesday of April, 1911.

---

THE Rochester Academy of Medicine held meetings during January as follows:

*Section of General Medicine.* Wednesday evening, January 4. Program: Some orthopedic and surgical indications in infantile paralysis, Albert H. Freiberg, Cincinnati, President American Orthopedic Association.

*The Annual Meeting* was held Wednesday evening, January 11.

*Section of Obstetrics, Gynecology, and Pediatrics.*—Wednesday evening, January 18. Program: Uterine retrodisplacements, Charles R. Barber.

---

THE Buffalo Academy of Medicine held meetings during December and January as follows:

*Section of Obstetrics and Gynecology.*—Tuesday evening, December 27. Program: When shall we operate in puerperal septic infection and, what can we expect from vaccines? John O. Polak, Brooklyn, N. Y.

*Section of Surgery.*—Tuesday evening, January 3. Program: Some common sources of errors in the diagnosis of renal and urethral calculi, Hugh Cabot, of Boston.

*Section of Medicine.*—Tuesday evening, January 10. Program: Low protein diet in kidney conditions, Horace D. Arnold, Boston.

